# Early Diagnosis of Low-Risk Papillary Thyroid Cancer Results Rather in Overtreatment Than a Better Survival

**DOI:** 10.3389/fendo.2020.571421

**Published:** 2020-10-06

**Authors:** Jolanta Krajewska, Aleksandra Kukulska, Malgorzata Oczko-Wojciechowska, Agnieszka Kotecka-Blicharz, Katarzyna Drosik-Rutowicz, Malgorzata Haras-Gil, Barbara Jarzab, Daria Handkiewicz-Junak

**Affiliations:** ^1^ Department of Nuclear Medicine and Endocrine Oncology, M.Sklodowska-Curie National Research Institute of Oncology Gliwice Branch, Gliwice, Poland; ^2^ Radiotherapy Department, M.Sklodowska-Curie National Research Institute of Oncology Gliwice Branch, Gliwice, Poland; ^3^ Department of Genetic and Molecular Diagnostics of Cancer, M.Sklodowska-Curie National Research Institute of Oncology Gliwice Branch, Gliwice, Poland

**Keywords:** papillary thyroid cancer, low-risk thyroid cancer, overdiagnosis, overtreatment, active surveillance, papillary thyroid microcarcinoma

## Abstract

We are witnessing a rapid worldwide increase in the incidence of papillary thyroid carcinoma (PTC) in the last thirty years. Extensive implementation of cancer screening and wide availability of neck ultrasound or other imaging studies is the main reason responsible for this phenomenon. It resulted in a detection of a growing number of clinically asymptomatic PTCs, mainly low-risk tumors, without any beneficial impact on survival. An indolent nature of low-risk PTC, particularly papillary thyroid microcarcinoma (PTMC), and the excellent outcomes raise an ongoing discussion regarding the adequacy of treatment applied. The question of whether PTMC is overtreated or not is currently completed by another, whether PTMC requires any treatment. Current ATA guidelines propose less extensive preoperative diagnostics and, if differentiated thyroid cancer is diagnosed, less aggressive surgical approach and limit indications for postoperative radioiodine therapy. However, in intrathyroidal PTMCs in the absence of lymph node or distant metastases, active surveillance may constitute alternative management with a low progression rate of 1%–5% and without any increase in the risk of poorer outcomes related to delayed surgery in patients, in whom it was necessary. This review summarizes the current knowledge and future perspectives of active surveillance in low-risk PTC.

## Introduction

Thyroid cancer is the most common endocrine malignancy. The vast majority of patients, more than 90%, are diagnosed with differentiated thyroid carcinoma (DTC). Among them, 89.8% have papillary thyroid carcinoma (PTC), 4.5% follicular thyroid carcinoma, and 1.8% Hurthle cell carcinoma. The remaining cases are diagnosed with medullary thyroid carcinoma (1.6%) or anaplastic thyroid carcinoma (0.8%) (1).

We are witnessing a rapid global increase in the number of detected thyroid carcinomas in the last thirty years. Its incidence, relatively stable until the early 1990s, has nearly tripled in the United States since 1975, from 4.9 to 14.3/100,000. Interestingly, an absolute increase in women was almost four times greater than in men. Similar data come from European registries (2–4). For example, in Switzerland, the age-standardized incidence of thyroid cancer increased from 5.9 to 11.7/100,000 in women and 2.7 to 3.9/100,000 in men (2). The most spectacular data come from South Korea, where thyroid cancer incidence changed in women from 10.6/100,000 in 1996 up to 111.3/100,000 in 2010 (5). Currently, thyroid carcinoma is the most common cancer in Korean women. It has been estimated that thyroid cancer will be the four most common malignancy by 2030 (6). However, this phenomenon concerns only PTC as the number of other histological types of thyroid cancer remains stable (2, 7).

Only 38% of thyroid cancers produce clinical symptoms such as a neck lump, throat and neck discomfort, swallowing difficulties, and rarely cough, voice change, dyspnea, or symptoms related to a metastatic disease that prompt patients to start diagnostics (8). Currently, the size of the detected PTCs decreased. Twenty-five percent of thyroid cancers, diagnosed between 1988 and 1989, had ≤ 1 cm in diameter, whereas 42% were larger than 2 cm. In 2008-2009, the number of diagnosed tumors > 2 cm was 33%, whereas the smaller ones ≤ 1 cm 39% (7). Small intrathyroidal PTC ≤1 cm represents between 23% and 51% of all thyroid cancers (8–12), identified mainly by neck ultrasound or found in at least 10% of thyroids removed for benign conditions (13). It has been demonstrated that even 71% of PMTCs were incidentally identified in histopathological material after thyroid surgery (14). PMTC was diagnosed in 2%–15.2% of cases of multinodular goiter (14).

Conversely, thyroid cancer-related mortality is somewhat stable (15) and ranges between 0.4 and 0.6 deaths/100,000 persons (16, 17) or even slightly decreased (2). The results of the most recent international age-period-cohort analysis showed that the long-term declines in thyroid cancer mortality were accompanied by decreases over calendar periods and birth control, indirectly confirming the critical role of overdiagnosis in thyroid cancer epidemy (17). However, according to the data concerning the last decade, a slight increase in the mortality rate from 0.4 per 100,000 person-years in 1994–1997 up to 0.46 per 100,000 person-years in 2010–2013 (18), first in men and next in women is noticed in the USA, the UK, some European countries (Spain, Germany, Italy), Australia, and in Japanese men (19). This increase in mortality rate is higher or restricted to patients above 40 years of age (19). Likewise, the number of incidentally detected DTCs in an autopsy carried out in patients died due to other reasons, without known thyroid pathology, has remained unchanged since 1970 (20). The prevalence of incidental DTC depends on the way of thyroid examination on autopsy. Based on the meta-analysis of 35 studies involving 12,834 autopsies, it was 4.1% for the partial examination and 11.2% for the whole examination of the thyroid gland (20). Similar data were provided by a systematic review of 15 papers, published between 1969 and 2005. Latent PTC was found in 989 out of 8,619 autopsies (21). The highest prevalence (35.6%) of occult PTC was found in the Finish population (22). These data clearly demonstrate not only the indolent nature of PTC but also prove the lack of meaningful health consequences related to these tumors.

Papillary thyroid microcarcinoma (PTMC) is a small thyroid malignancy measuring ≤1 cm. An intrathyroidal PTMC without nodal and distant metastases is recognized as a very low-risk thyroid carcinoma. The tumor diameter is frequently less than 2 mm, as observed in 60% of patients (13). However, according to other data, it may range from 4.1mm to 8 mm in 35.2%–79% of cases (14). Such lesions are usually detected either by imaging procedures or through a histopathological examination of the thyroid gland operated due to benign disease. A meaningful percentage of the occult PTMC (33%–79%), found in autopsies, was smaller than 1 mm. Twenty-seven up to fifty percent were multifocal lesions (13), whereas extrathyroidal extension was observed in 25.7% of cases (14). Lymph node metastases occurred in 4.4% of patients with tumor diameter ≤5 mm. This percentage was a bit higher in tumors >5 and >8 mm. Lymph node metastases at diagnosis also correlated with nonincidental tumors, extracapsular invasion, the follicular PTC variant, or presence of Hashimoto thyroiditis (14). Similarly, according to an analysis of 1,066 patients, the male gender, younger age ≤ 45 years, multifocality, extrathyroidal extension, and larger primary tumor size > 6 mm were risk factors for lymph node metastases (23). Distant metastases at initial presentation occurred in a meager percentage of patients—0.37% (14).

PTMC represents an indolent type of thyroid carcinoma characterized by a very low mortality risk of less than 1% after 20 years following thyroid surgery (24). No differences in mortality risk were noticed between PTMC patients with incidental and non-incidental thyroid tumors (25). More importantly, no deaths were reported in nontreated patients subjected to active surveillance programs (26–28). The risk of recurrence is also extremely low, ranges between 1% and 5%, regardless of the presence of palpable lymph nodes at diagnosis. Extrathyroidal extension, large lymph node metastases ≥ 2 cm, and poorly differentiated component were risk factors of unfavorable outcomes in symptomatic PTMC (29). This risk is even lower <1% if palpable lymph nodes are excluded (13). Data obtained in a meta-analysis, published in 2008, showed the risk of local/nodal recurrence of 2.4% (231 out of 9,379 analyzed patients), whereas distant metastases were reported in 26/9,379 cases (0.27%) (14). The risk of local recurrence was significantly higher in younger patients (< 45 years), in clinically overt carcinomas, in case of multifocal tumors and the presence of lymph node metastases at diagnosis (14). According to another systemic review and meta-analysis comprising 854 patients with incidental PTMC and 2,669 patients with non-incidental PTMC it was demonstrated that the recurrence rate was significantly lower for incidental tumors (0.5%) compared to non-incidental ones (7.9%) (30). However, nor age, neither sex, size, tumor multifocality, lymph node involvement, and treatment modality were considerably associated with disease relapse (30).

## Is Overdiagnosis the main Reason of a Growing Incidence of Low-Risk PTC?

Cancer overdiagnosis, by definition, “occurs if the disease is diagnosed correctly, but the diagnosis produces an unfavorable balance between benefits and harms” (31). One shall distinguish an overdiagnosis from a false-positive result. According to Carter and Barratt, a false positive diagnosis concerns a patient who is incorrectly informed that may have cancer (31). The question of whether we face the apparent or true increase in thyroid cancer incidence is still open. The analysis of new DTC cases registered in the SEER database, diagnosed between 1988 and 2005, showed an increase not only of PTMCs but also of more advanced tumors > 4 cm in diameter and those with distant metastases (15). Similar data were obtained for children, adolescents, and young adults. Significant increasing trends were noticed for small PTCs <5 mm, 5–9 mm, 10–19 mm, as well as for tumors larger than 20 mm (32). Such findings may speak for a true increase. One of the most recent analyses demonstrated an increase in PTC incidence during 2000–2016 in women aged 20–29 years and during 2000–2012 in women aged 30–39 years, whereas between the years 2012–2016, PTC incidence stabilized in a group aged 30–39 years or even decreased in a group aged 40–49 years. There were ethnic/racial differences in the analyzed groups, with the highest incidence in white and Hispanic women aged 20–29 years. (33). The authors concluded that better diagnostic scrutiny was not probably the only cause of the growing number of PTC cases. Racial disparities with the highest incidence among white persons were also reported by the Marcadis group (34).

As the thyroid cancer increase almost exclusively concerns PTC, Pellegriti et al. (35) considered a possible role of specific carcinogens that might favor certain molecular abnormalities, characteristic for PTC. Such a hypothesis is reflected by an increase in the number of BRAF (B-Raf-proto-oncogene, serine/threonine kinase)-mutant PTC (35). The frequency of BRAF-positive PTC differs in distinct geographical localizations, so, a potential role of dietary factors or chemical compounds has to be considered (36, 37). A two-fold higher risk of *BRAF* mutation was noticed in regions with high iodine concentration in drinking water compared to those of normal iodine concentration (38). Similarly, regarding the follicular PTC variant, an increase in the frequency of *RAS* (Rat sarcoma viral oncogene homolog) mutations was observed.

Radiation exposure due to extensive use of X-rays in medical and dental diagnostic procedures, particularly in younger age, iodine intake, different environmental factors such as pollutants, high concentration of boron, vanadium, manganese, and iron in volcanic regions, chemical factors including polybrominated diphenyl ether flame retardants (PBDEs), viruses (herpes, Epstein-Barr virus), body weight and insulin resistance, TSH level, or estrogens are among factors definitely or potentially contributed to thyroid cancer incidence (35, 36, 39).

Radiation exposure is the only factor, which role in thyroid carcinogenesis was clearly demonstrated. Depending on the modality and severity of irradiation, the thyroid damage may lead to cell death or, if less severe, may cause specific genetic abnormalities leading to thyroid carcinogenesis. (40). The thyroid is very radiosensitive, particularly at a young age, so there is an inverse relationship between the age at radiation exposure and thyroid cancer risk (41). First data demonstrating a negative impact of medical radiation exposure on the thyroid, leading to an increased risk of thyroid carcinoma, were reported in 1950. Significant growth in thyroid cancer incidence in childhood was observed after the Chernobyl Nuclear Power Plant Accident (39, 40). However, radiation exposure is not likely to be a cause of the PTC epidemic as the percentage of *RET-PTC* rearrangements, believed to be a molecular fingerprint of radiation-induced PTC, decreased (12).

The impact of other factors is not so definite and requires further studies. The role of iodine intake, although it changed the epidemiology of DTC, is still discussed. Based on available data, Zimmerman and Galetti suggested iodine deficiency as a risk factor for follicular thyroid cancer and possibly for anaplastic thyroid cancer. However, the question regarding the causal role of iodine intake in PTC is equivocal. In many countries, the introduction of iodized salt was related to an increase in PTC incidence, whereas in some countries, including Australia, the US, and Switzerland, the number of new PTCs grew despite stable or decreasing iodine intake (42). Other micro- and macronutrient factors may influence the risk of the development of thyroid cancer. Some data pointed out the relationship between thyroid cancer incidence and obesity, carbohydrate, or excess in carbohydrate accumulation, or protein consumption (35, 36, 39).

Xenobiotics, the exogenous compounds and chemicals, may also interfere with thyroid function and thereby may change DTC risk. Such so-called “endocrine disruptors” may act as competitive inhibitors of the sodium/iodide symporter (perchlorate and nitrate), inhibit thyroperoxidase (TPO) activity (isoflavones), inhibit binding of thyroid hormone to transport protein (polybrominated diphenyl ethers; PBDE), bind to thyroid hormone receptors (PBDE and bisphenol A; BPA), inhibit peripheral deiodinase activity (styrenes), or decrease the half-life thyroxine (T4) in serum (PBDE and dioxins). The exposition to these compounds may result in a decrease in serum T4 concentration and an increase in serum TSH levels. However, the question of whether they promote carcinogenesis remains open (35, 39). The polluted atmosphere, water, and soil are among other environmental factors, which role in thyroid cancer development has to be considered. The data published by Drozd et al. suggested that nitrate pollution in drinking water may modify the risk of radiation-induced childhood thyroid cancer in Belarus (37). An increased exposition to hexachlorobenzene in Spanish people leaving near an organochlorine factory was probably the reason for a high incidence of thyroid cancer (39). Another question is related to a higher risk of thyroid cancer in volcanic areas, among others in Sicily, Iceland, French Polynesia, or volcanic islands of Hawaii (36, 39). A high concentration of boron, vanadium, manganese, iron, fluorine, selenium, and different trace elements in volcanic regions act as endocrine disruptors, influence the function of the thyroid gland and may promote thyroid cancer (43). The data on the role of tobacco smoking, although it is a well-recognized risk factor in numerous malignancies, are conflicting. Some of them demonstrated even an inverse relationship between DTC and cigarette smoking (39).

The discrepancy between a growing number of low-risk PTCs and a stable number of latent PTC found in autopsies indicates that this high incidence is related to more accurate and easily accessible diagnostic methods than reflects a real trend (4, 7, 11, 44, 45). Palpable thyroid nodules could be detected in 3%–7% of the world population. This percentage increases even up to 76% if ultrasonography is used as it is able to found lesions as small as 2 mm (36, 46). Tumors ≤ 1 cm in diameter were associated with older age (≥45 years), female sex, and a higher probability of being detected with thyroid ultrasound (9). According to the Medicare data, the number of thyroid ultrasound as initial imaging per 100,000 people has increased over time at a rate of 20.9% per year between 2002 and 2013 (10). A significant relationship between the use of thyroid ultrasound and PTC incidence in particular areas has been demonstrated (10). Clinically asymptomatic thyroid nodules are also accidentally found in up to 25% of contrast-enhanced chest computed tomography (CT), 16%–18% of neck CT or MRI (magnetic resonance imaging), and in 1%–2% of fluorodeoxyglucose (FDG) PET (positron emission tomography) scans (47).

The patients more frequently tested by routine medical procedures are diagnosed with more thyroid cancers (48). Interestingly, it is more probable that screening programs detect slow-growing and indolent tumors than fast-growing and aggressive cancers. The time intervals between diagnostic procedures, based on the best cost-benefit ratio and least harm, are too long for aggressive tumors. On the contrary, fast-growing cancers producing symptoms are more likely to prompt a patient to contact a doctor any time (31). It has been estimated that about 11,000 cancers in women and 18,000 cancers in men are overdiagnosed in Australia each year (49). Interestingly, the screening program introduced in Fukushima after the nuclear plant accident showed thyroid cancer incidence 30 times higher than the national incidence in children and adolescents both in contaminated and non-contaminated regions (13). National screening procedures in South Korea were responsible for thyroid cancer incidence more that was 15 times greater than that in the UK and 5.6 times that of the USA (11, 50). Increasing use of thyroid sonography in the US between 2003 and 2013 detected at least 6594 thyroid cancers in adults ≥ 65 years. Patients with one or two comorbidities were more likely to be diagnosed with thyroid cancer as they more frequently had neck ultrasound (10).

Improvement of diagnostic accuracy of thyroid sonography may help to reduce the number of unnecessary fine-needle aspiration biopsies (FNAB). The goal of the recent analysis of 502 thyroid nodules was the comparison of five commonly used risk-stratification systems: American College of Radiology Thyroid Imaging Reporting and Data System (ACR TIRADS), American Thyroid Association (ATA) risk stratification, American Association of Clinical Endocrinologists (AACE), Korean Society of Thyroid Radiology (K-TIRADS) and European TIRADS (Eu-TIRADS). It was demonstrated that application of these systems resulted in the reduction of FNAB number by 17.1% up to 53.4%. The ACR TIRADS was characterized by the highest negative predictive value of 97.8%, with a false negative ratio of 2.2%. It allowed for the largest reduction of unnecessary FNABs (51).

Extensive implementation of cancer screening may lead not only to its overdiagnosis but also may exert, among others, a significant financial health-care impact as one of its consequences is an overtreatment. So, a possible way to resolve this problem is to reduce the number of new cases diagnosed, particularly very low-risk PTMC. It is reflected in the current ATA guidelines. Thyroid sonography with the assessment of cervical lymph nodes is recommended in all patients with known or suspected thyroid nodules but not as a screening procedure (52). The indications for FNAB, in turn, are limited to thyroid nodules, larger than ≥ 1 cm in the greatest dimension. What is more important, not all thyroid nodules ≥1 cm require FNAB. FNAB is recommended in nodules ≥ 1 cm only if high-risk (strong recommendation, moderate-quality evidence) or intermediate-risk (strong recommendation, low-quality evidence) sonographic features are present. If nodules have with low or very low-risk sonographic pattern, the biopsy is recommended in tumors ≥1.5 or ≥2 cm, respectively (52). It substantially differs from the Japanese attitude. At Kuma Hospital, they believe that it is better to diagnose a suspicious small nodule and discuss the FNAB result with a patient (26). Their idea is to avoid unnecessary treatment when patients contact another physician less familiar with thyroid carcinoma.

## Overtreatment Does Not Result in Better Outcomes

An indolent nature of the low-risk PTC, particularly PTMC, and the excellent outcomes, including data coming from first reports on active surveillance, raise an ongoing discussion, regarding the adequacy of treatment applied. The question of “whether PTMC is overtreated or not” is currently completed by another, “whether PTMC requires any treatment”. The current ATA guidelines (52) comparing to the previous one (53) meaningfully move PTC management toward a less aggressive approach. In 2009 ATA recommended total or near-total thyroidectomy in all DTC cases > 1 cm in diameter unless there were no contraindications for such procedure. Thyroid lobectomy alone was considered as sufficient treatment only in patients with small (<1 cm), unifocal, intrathyroidal PCA in the absence of prior head and neck irradiation or radiologically or clinically evident lymph node involvement (53). In 2015, total thyroidectomy was definitely recommended only for patients with thyroid cancer >4 cm, or if gross extrathyroidal extension, clinically apparent lymph node involvement or distant metastases are present. Patients with lower local advancement, including DTC >1 and <4 cm, clinically N0, without extrathyroidal extension, may be subjected to either bilateral (total or near-total thyroidectomy) or unilateral one (thyroid lobectomy) surgical procedure (52). Regarding PTMC, the guidelines use the statement “*if surgery is chosen* for thyroid cancer <1cm without extrathyroidal extension and cN0, the initial surgical procedure should be a thyroid lobectomy unless there are clear indications to remove the contralateral lobe”. Although there is no recommendation, which defines the criteria for active surveillance, such a statement may be cautiously interpreted that ATA considers it as an option for PTMC. One may expect a more precise guideline in the next update as ATA listed active surveillance among the directions for future research.

Following the increase in thyroid cancer incidence, a growing number of thyroidectomies is observed (2). It was demonstrated that 38.2% of 2,563 patients subjected to surgery had thyroid cancer ≤1 cm in diameter (9). Moreover, more that 50%, even up to 80% of patients operated due to low-risk PTC, including tumors ≤ 2 cm in diameter, still undergo total thyroidectomy, although the risk of death from small PTC is extremely low and not influenced by the extent of surgical procedure (lobectomy vs. total thyroidectomy) (54–56). Even for larger PTC tumors, between 1 and 4 cm in adults, there were no differences in overall survival depending on the surgical approach (total thyroidectomy vs. lobectomy) (57). However, in this study, patients subjected to total thyroidectomy more frequently had multifocal tumors, extrathyroidal extension, lymph node involvement, distant metastases, positive surgical margins, and received radioiodine. Similarly, the risk of recurrence in PTMC without lymph node metastases at presentation does not differ between patients after lobectomy and total thyroidectomy (13).

The analysis of 29,512 PTMC patients, based on the SEER registry (1998–2010), does not show any significant differences in disease-specific survival between patients who underwent partial or total thyroidectomy. What is more important, PTMC patients subjected to any thyroid surgery had similar overall survival to the USA general population (58). Another study, also based on the SEER registry data, involved PTC staged T1-4N0M0, stratified by nonsurgical (1,453 patients) and surgical management (54,718 patients). Regrading younger patients (aged between 14 and 55 years) surgical and nonsurgical approach did not significantly differ in the 10-year disease-specific survival among 0–4cm PTCs, 4.1–6 cm PTCs, or even among tumors larger than 6 cm (59). Although indications for surgery in patients with larger PTCs do not seem disputable, these data clearly reflect an indolent nature of PTC. There are some published data demonstrating that patients who underwent total/near-total thyroidectomy and lymph node excision had a lower risk of recurrence. However, these differences were not significant (14). Such data raise the question of whether surgical treatment is necessary for PTMC patients?

If no other adverse features are present, radioiodine (RAI) is not recommended after lobectomy or total thyroidectomy for PTMC patients. Considering low-risk PTC patients, ATA does not routinely recommend RAI remnant ablation. Individual patient features, patient preferences, and disease follow-up implications should be involved in a decision-making process (52). Such a position results from the lack of EBM (evidence-based medicine) proofs confirming a beneficial impact of postoperative RAI treatment on disease-free and overall survival in low-risk PTC. However, this recommendation is weak and based on low-quality evidence. The recent analysis, published in 2019, which involved 32,229 PTC patients, among them 17,286 low-risk cases, demonstrated that nearly 25% (4,300) of low-risk cases still undergo RAI remnant ablation in the USA. Patients who were given RAI were younger (mean age 49.9 years), more often Hispanic or Asian, and more often insured than those who did not receive RAI. Moreover, patients treated with RAI more frequently had a total thyroidectomy and lymph node resection. Noteworthy, no patient treated with RAI died during the follow-up, whereas in the non-treated group—0.04% (5 patients) (55). Even a substantial percentage of PTMC patients (14,146 out of 60,586 analyzed cases) received postoperative RAI therapy according to the American National Cancer Data Base. These patients more often had a multifocal disease, larger tumors, Hispanic origin, low income, and were treated in non-academic centers. However, along with the increase of PTMC incidence, the number of PTMC or low-risk PTC patients treated with RAI decreased (60, 61). Currently, the probability of RAI administration in patients with localized PTC is lower if the patients are older > 65 years, had tumors <1 cm, and when they are treated in academic centers (61).

## Active Surveillance—Is it a Proper Direction for PTMC?

Active surveillance consists of close monitoring of cancer without initial surgery or other treatment. One shall notice the difference between active surveillance and watchful waiting, which primarily is based on symptom management in patients who are likely to die due to other reasons (62). PTMC represents a large reservoir of subclinical thyroid cancer. According to Leboulleux et al. occult PTMC may “affect roughly 20 million adults in the USA and 48 million in Europe” (13). It is unlikely that its indolent biological behavior, discussed above, will change over time. Thus, the idea of active surveillance seems to be an option for very low-risk small PTCs.

The history of active surveillance started more than 25 years ago in Japan. Based on a high incidence of latent thyroid cancer found in autopsies and on the results of screening procedures detecting thyroid carcinoma in 3.5% of otherwise healthy Japanese women, Dr. Akira Miyauchi hypothesized that the majority of low-risk PTCs did not progress at all or showed a very slow progression. He assumed that 1. active surveillance could identify a small percentage of PTMC that progressed and developed lymph node metastases, 2. delayed surgery for these PTMC did not result in poorer outcomes, 3. surgical management applied in all PTMCs did more harm than good (26).

The data on active surveillance in low-risk and very low-risk thyroid carcinoma published so far are encouraging (**Table 1**). The first retrospective report came from the Kuma Hospital in Japan, where the idea of active surveillance in thyroid carcinoma was introduced (63). One hundred sixty-two out of 732 patients diagnosed with PTMC between 1993 and 2001 chose follow-up without surgery. More than 70% of nodules remained unchanged. Only 10.2% increased by more than 10 mm, whereas lymph node metastases in the lateral neck compartment occurred in 1.2% of patients. Regarding 626 operated patients, 570 persons chose surgery at diagnosis, and the remaining 56 were operated after the follow-up period. Lymphadenectomy was performed in 594 patients, and, what is surprising, lymph node metastases were diagnosed in 50.5% of them. Multifocal tumor growth was observed in 48.2% of patients. The rate of recurrence in this group was 2.7% after 5 years and 5.0% after 8 years (63). Subsequent retrospective analysis, carried out at the Kuma Hospital between the years 1993 and 2004, was published in 2010. In this paper, the outcomes of 340 PTMC patients subjected to active surveillance were compared to 1,055 patients who underwent immediate surgery. The risk of 5 and 10-year tumor enlargement by 3 mm was 6.4% and 15.9%, respectively. Lymph node metastases occurred in 1.4% of patients after 5 years and in 3.4% of patients after 10 years. The mean follow-up was 74 months (range 18–187 months). No factors related to the tumor increase or nodal metastases were found. None of the patients after delayed surgery showed PTC recurrence (27). The latest summary of active surveillance at the Kuma Hospital was published in 2014. It reported a retrospective follow-up (mean time 60 months; range 18–228) of a group of 1235 PTMC patients, who chose the observation. Only 58 (4.6%) patients showed the enlargement of the nodule. The number of patients who developed lymph node metastases was lower −19 (1.5%). The percentage of patients with progression was lowest in older patients. Age < 40 years and tumor size 9 mm or larger were independent factors related to disease progression. Importantly, there were no distant metastases or cancer-related death under this study (28).

**Table 1:** Summary of the results of studies on active surveillance presented in this review.

Study data	Size of thyroid nodules included	Number of patients subjected to AS	Disease progression criteria	Mean follow-up	Percentage of patients with tumor progression	Percentage of patients with LN metastases
Retrospective studies						
Ito (Japan) (63)	PTMC	162	Nodule increase ≥2 mm	47 months (18–113)	10.2%	1.2%
Ito (Japan) (27)	PTMC	340	Nodule increase by ≥3 mm; development of LN metastases	74 months (18–187)	6.4%* 15.9%**	1.4%* 3.4%**
Ito (Japan) (28)	PTMC	1,235	Nodule increase up to 12 mm or more; development of LN metastases	60 months (18–228)	4.6%	1.5%
Prospective studies						
Sugitani (Japan) (29)	PTMC	230 (300 nodules)	Nodule increase ≥3 mm; invasion of local structures; development of LN or distant metastases	5 years (1–17)	7%	1%
Tuttle (USA) (64)	PTC ≤ 15 mm	291	Nodule increase ≥3 mm; extrathyroidal extension; invasion of local structures; development of nodal or distant metastases	25 months (6–166)	3.8%	0%
Sakai (Japan) (65)	PTC T1bN0M0	61	Nodule increase ≥3 mm; development of nodal or distant metastases	7.4 years (0.5–25)	7%	3%
Molinaro (Italy) (66)	Bethesda V (suspicious for PTC) or VI (PTC) nodules ≤13 mm	93	Nodule increase by ≥3 mm; development of LN metastases	19 months (6–54)	2%	1%

PTC, papillary thyroid carcinoma; PTMC, papillary thyroid microcarcinoma; LN, lymph nodes; AS, active surveillance; *5-year follow-up; **10-year follow-up.

Prospective data concerning active surveillance are scarce. After the observation of 300 asymptomatic PTMC at the Cancer Institute Hospital in Tokyo for a mean of 5 years (range 1–17) 269 tumors (90%) remained unchanged, 9 (3%) decreased, whereas the remaining 22 (7%) increased in size. Three patients (1%) with lymph node metastases and nine (4%) with tumor increase underwent surgery. Nobody had an extrathyroidal extension or developed distant metastases, as well as no postoperative recurrences, were diagnosed. On the contrary, 10-year cause-specific survival in a group with symptomatic PTMC was only 80%. (29). The authors did not clearly define which patients were considered symptomatic. One may guess that term “symptomatic PTMC” concerned patients with lymph node or distant metastases or extrathyroidal invasion confirmed by imaging studies. In another analysis, carried out in a group of 291 low-risk PTC patients with intrathyroidal tumors ≤15 mm, nodule progression by 3 mm or more were stated in 11 (3.8%) patients, with a 5-year cumulative incidence of 12.1%. No lymph node or distant metastases occurred in this group. The mean follow-up was 25 months (range 6–166 months) (64). Recently, the cut-off value of the PTC diameter qualified for active surveillance has moved toward pT1bN0M0 tumors. Japanese researchers reported the results of a prospective analysis in a group of 392 T1bN0M0 PTC patients, among whom 61 persons chose active surveillance over surgery, and compared them to 331 patients who underwent surgery. Tumor diameter ranged from 11 to 16 mm. After a mean observation of 7.4 years, four (7%) T1bN0M0 tumors increased in size, whereas two patients (3%) developed lymph node metastases. Weak calcification and rich vascularity were risk factors for tumor growth, while younger age was a predictor for lymph node metastasis (65).

First European experiences with active surveillance in PTMC come from Italy. Ninety-three patients with a thyroid nodule ≤1.3 cm and cytological diagnosis of PTC (Bethesda VI) or suspicious for PTC (Bethesda V) were enrolled in a prospective observational study with the median duration of follow-up of 19 (range 6–54) months. Clinical or sonographic evidence of extrathyroidal extension, lymph node or distant metastases, and hyperthyroidism were among the key exclusion criteria. Twenty patients dropped out of the study for personal reasons. Three percent of patients (3/93) showed PTMC progression, among them two persons—tumor increase by more than 3 mm in each dimension, and one patient lymph node metastases. All of them were diagnosed with the Bethesda V category. At the time of data cut-off, 71 patients (76%) were still in follow-up (66).

A systematic review and a meta-analysis (67), published in 2019, was based on the evaluation of 6 studies, including four presented above (27, 28, 64, 65). The pooled proportion of the tumor diameter increase and lymph node metastasis at 5 years was 5.3% and 1.6%, respectively (67). The most recent meta-analysis included 9 papers, 4,156 patients at a mean age ranged from 51 to 54 years, mainly females, among them 3,120 persons from Japan, 688 from South Korea, 291 from the United States, and 57 from Colombia. Searching criteria included relevant studies of active surveillance for low-risk PTC defined as T1a or T1b N0M0. Seven studies enrolled patients with tumors ≤ 10 mm, while the remaining two expanded size criteria up to 15 mm. The pooled data demonstrated the risk of tumor growth to be 4.4%, whereas metastatic spread to cervical lymph nodes – 1.0%. Thyroid cancer-related pooled mortality was 0.03%. The pooled percentage of delayed surgery, reported in 8 out of nine analyzed studies, was 9.9%. However, patient preference but not a disease progression was the main reason for surgery—51.9%. The recurrence rate after a delayed surgery was 1.1% only (68). Such data clearly demonstrate that active surveillance in low-risk PTC may be a safe option for the patients and does not produce any substantial risk related to a delayed surgery, in patients in whom it was necessary. We have to wait for the outcomes of other ongoing clinical trials. We may expect new EBM data sufficient to introduce active surveillance into daily clinical practice in the USA and Europe.

Younger age, mainly below 45 years, was a factor associated with PTC progression in some studies on active surveillance (28, 64, 65, 69, 70), whereas in other not (71, 72). Some sonographic features may help to predict PTC progression during active surveillance, among them weak calcification and rich vasculature (65). Kim et al. reported that sustained, high mean TSH level, above the cut-off point of 2.5 mU/L, was associated with PTMC progression during active surveillance (69). However, the earlier report did not find any significant correlation between the mean TSH level and PTMC progression (72). Hirokawa et al. analyzed pathological features of PTMC tumors that demonstrated progression or developed lymph node metastases during active surveillance ≥ 1 year. The values of Ki67 indices were above 5% and 10% in 50% and 22% of growing tumors, respectively. Simultaneously, these values were significantly higher than those observed in non-progressing tumors. Intraglandular dissemination and psammoma bodies, in turn, correlated with the occurrence of lymph node metastases (70).

**Figure 1:**
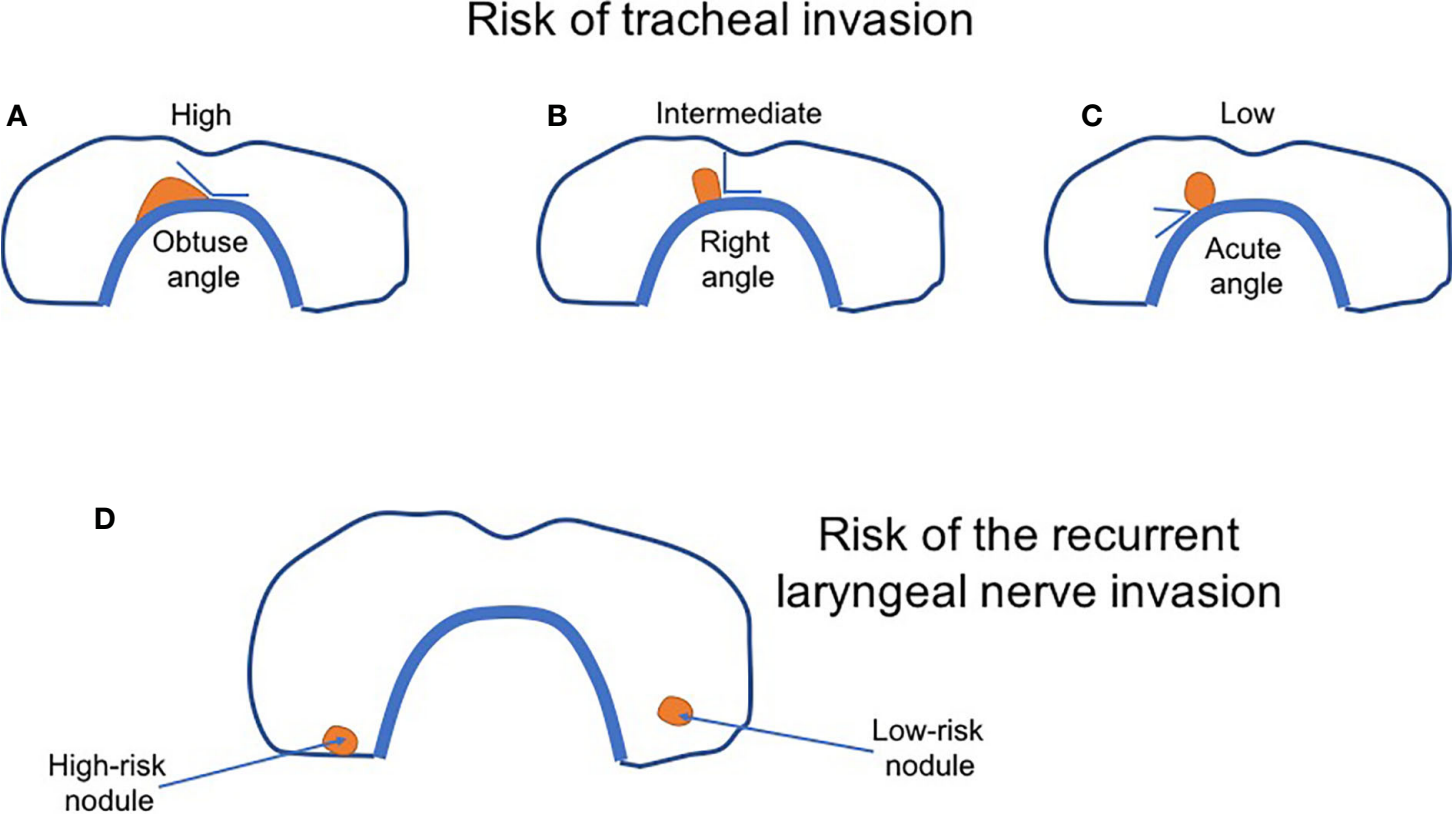
The scheme of sonographic classification of thyroid nodules based on the risk of tracheal and the recurrent laryngeal nerve invasion. **(A–C)** The risk of tracheal invasion depends on the angle between the nodule and tracheal wall. **(A)** High-risk nodule—an obtuse angle; **(B)** Intermediate-risk nodule—a right angle; **(C)** Low-risk nodule—an acute angle. **(D)** The risk of the laryngeal nerve invasion. Low-risk nodule (right) is surrounded by a thin of the normal thyroid. High-risk nodule (left)—no rim of the normal thyroid is observed. A nodule closely adheres to thyroid capsule and the nerve. The figure was modified by the authors based on Akira Miyauchi paper (67).

Considering active surveillance, one should define its protocol, determine the duration of monitoring, identify who is an appropriate candidate, and finally, its effect on the patient’s emotional health (62). One of the most critical issues is to accurately define a candidate for active surveillance to avoid overtreatment on the one hand and simultaneously not to produce any increase in the risk of cancer-related mortality. It seems evident that not all low-risk PTC patients should be subjected to active surveillance. Miyauchi et al. listed several contraindications, among them the presence of lymph node and distant metastases, suspicion of high-grade malignancy on FNAB, and some sonographic features. When the nodule is located next to the recurrent laryngeal nerve or attaches the trachea at an obtuse angle, such patients should be referred to surgery (26, 73) (**Figure 1**). On the contrary, a nearly right angle was associated with an unclear or moderate risk, whereas an acute angle between the tumor and trachea with a low risk (73). The risk of the laryngeal nerve invasion was greater in the absence of a normal thyroid rim close to the nerve location (73). However, according to the Japanese researchers, multifocal tumors and a positive family history of non-medullary thyroid carcinoma did not exclude from active surveillance (26).

In 2010 three distinct types of PTMC were proposed. Type I represented harmless, asymptomatic, incidentally identified the lowest risk PTMC. Type II involved an early stage of usual low-risk PTC, whereas type III comprised of clinically apparent high-risk PTMC. The choice of treatment strategy varied depending on the tumor type. Type I tumors qualified for active surveillance, type II for lobectomy when increasing size was observed, and type III for more aggressive treatment with total thyroidectomy and RAI (29). Unfortunately, no precise criteria for each type were given. Such a risk-stratified approach should evaluate three interrelated but distinct domains, including tumor/neck ultrasound characteristics, patient characteristics, and finally, medical team characteristics. Tumor/neck ultrasound characteristics has to consider primary tumor size, its location within the thyroid, molecular profile, and the status of cervical lymph nodes. Regarding patient characteristics, age, child-bearing potential, family history of thyroid cancer, comorbidities, patient’s willingness to defer immediate surgery, and compliance with follow-up are among the factors for consideration. Medical team characteristics, in turn, is based on the availability and expertise of the multidisciplinary team, the quality of sonography, and the experience of the treating physician (74). Based on these domains and the data presented above, an ideal candidate would be an older patient, with a probable or proven solitary PTMC, characterized in sonography by a well-defined margin, confined to thyroid parenchyma, and not adjacent to the thyroid capsule. Younger patients, with multifocal disease, tumor adjacent to the thyroid capsule in noncritical locations, potentially more aggressive phenotype, or the presence of other sonographic features that make follow-up difficult (thyroiditis, non-specific lymphadenopathy, etc.) are considered as inappropriate candidates. Finally, patients with tumors showing critical subcapsular location (adjacent to the recurrent laryngeal nerve or trachea) with the evidence of extra thyroid extension, metastases, or progression on serial examinations are inappropriate candidates (74).

Currently conducted a Multicenter Prospective Cohort Study of Active Surveillance on PTMC (MAeSTro study) involves adult patients, with a thyroid nodule ≤ 1 cm, diagnosed by fine-needle aspiration biopsy as Bethesda V (suspicious for thyroid cancer) or Bethesda VI (thyroid cancer). Multifocal tumors or a positive family history of non-medullary thyroid carcinoma are allowed. The infiltration of adjacent structures (trachea, esophagus, major vessels, nerves, or muscles), suspicious lymph nodes, poorly differentiated histology or a higher risk PTC variant, and Graves’ disease with an indication for RAI treatment or surgery are the main exclusion criteria (75). A Canadian prospective observational study of decision-making on active surveillance for low-risk PTC consider adult patients as eligible if they have PTC ≤ 2 cm in diameter, confined to the thyroid and simultaneously not adjacent to trachea or the recurrent laryngeal nerve, without lymph node involvement, or the diagnosis of poorly differentiated or non-papillary thyroid cancer (76, 77).

Although the molecular profile of the nodule may be helpful in the risk stratification, there are no currently validated molecular risk factors used in the decision-making process qualify for active surveillance to differentiate between an early stage aggressive lesion and an indolent occult cancer (74). *BRAF* mutations are commonly observed in PTMC, with a frequency ranging between 0% and 92.6%, on average, 57.4% (78). Importantly in some papers, their occurrence in PMTC did not significantly differ from PTC. In a Korean study, it was 65.5% and 67.2%, respectively (79). However, other reports demonstrated a higher percentage of *BRAF* mutations in PTC (78). *BRAF* mutations were more prevalent in classic (43%–81% of cases) and tall cell (93%–100% of cases) PTMC variants than in follicular one (0–67% of cases) (78). According to the most recent study, published by these Korean researchers, the frequency of *BRAF* mutation was higher in PTMC > 0.5 cm than in smaller ones ≤ 0.5 cm and decreased again in PTCs > 2 cm (80). Such a high prevalence speaks against using the *BRAF* mutation as an independent prognostic or predictive factor. However, Kim and coworkers believe that BRAF status may facilitate choosing the candidates for active surveillance. Their most recent multicenter study that included 743 PTMC patients, treated with total thyroidectomy, showed a significantly higher risk of tumor recurrence in BRAF positive group than BRAF negative one, 10.8% vs. 6.4%, respectively (81). These data are concordant with a meta-analysis, which involved 2,247 PTMC patients and demonstrated an increased risk of recurrence in BRAF positive tumors (82). On the contrary, in Yabuta study, described below, the *BRAF* mutation was not a predictive factor in term of tumor behavior as in was present in 64%, 70%, and 80% of PTMC from the non-progressing group, size-increase group, and lymph node metastasis group, respectively (83).

Yabuta and coworkers analyzed the risk of PTMC progression on active surveillance with reference to the *TERT* promoter mutations. They selected three groups of patients: the non-progressing group, the size-increase group, and the lymph node metastasis group. They did not find *TERTp* mutations in any of the analyzed groups (83). However, such mutations may be incidentally present in PTMC (84), even in tumors <5 mm in diameter (80), without any significant correlation with unfavorable clinical features (85, 86).

Rodrigues and coworkers concluded their comprehensive review regarding the molecular biology of PTMC, based on currently available data, nor the *BRAF* mutation alone, neither any other molecular alternation is sufficient to predict an aggressive behavior of PTMC (78). We share their opinion. Similarly, there are also no unequivocal clinical data. Further prospective studies are necessary to resolve this question.

Another important point is to define the protocol of active surveillance. At Kuma Hospital, ultrasound examination is repeated 6 months after the initial one, and next once a year (26). Sugitani et al. proposed a follow-up with ultrasonography every 6 or 12 months. MAeSTro study started in June 2016, scheduled follow-up visits every six months during the first two years, and every year thereafter (75).

The definition of tumor progression is not less important. Kuma Hospital proposes a tumor increase by > 3 mm or the development of lymph node metastases (26). According to the MAeSTro study, an increase in the longest tumor diameter by at least 3 mm, or ≥ 2 mm in two dimensions, suspected organ involvement, or the occurrence of lymph node/distant metastases fulfills the criteria of disease progression. Similar criteria of disease progression are proposed by the Canadian study, mentioned above. An additional criterion concerns PTC growth in a location adjacent to the trachea or the recurrent laryngeal nerve (76). Tuttle et al. demonstrated that three-dimensional measurement of the tumor volume allowed for the identification of the tumor progression with the median of 8.2 months (range 3–46 months) earlier than an increase in a single diameter (64). Changes in tumor volume were also evaluated in a retrospective Korean study, carried out in 192 PTMC patients. Seventy-two patients showed an increase in tumor volume more than 50% without an increase of the longest tumor diameter ≥ 3 mm. This finding confirmed the observation published by Tuttle group. Only four patients from the Korean group had both an increase in tumor volume and in the longest dimension ≥3 mm (71).

## Quality of Life, Psychological, and Economic Considerations

The diagnosis of malignant neoplasm is not only an essential medical event, but it also concerns different economic, psychological, and sociological issues. It is of particular relevance in some malignancies, showing a growing incidence. Early detection does not result in any improvement of their curability, like in non-invasive breast, thyroid, and prostate carcinoma (49), leading to overdiagnosis. Patients overdiagnosed with thyroid cancer are harmed by the psycho-social aspect of cancer diagnosis, treatment applied, and treatment-related consequences. Rogers et al. defined ethical concerns related to thyroid cancer overdiagnosis. The way we informed a patient about the disease, treatment modalities, and possible risk is of particular relevance. We are obliged to give objective data, respect patient’s autonomy, promote patient’s wellbeing, avoid harm, and consider matters of justice. One should consider pain, inconvenience, and anxiety at diagnosis, short and long-term impact of therapeutic approach (postoperative transient and permanent complications, postoperative follow-up, complying with medications), worry about recurrence, threats to personal identity, social participation and relationships. Surprisingly, a thyroid cancer diagnosis may result in a significant financial disadvantage as in the USA, patients diagnosed with any cancer had a higher bankruptcy rate than people without malignant neoplasms. Other points are a considerable economic impact on health care and various conflict of interest (87).

Active surveillance has been implemented for managing low-risk prostate cancer for many years. It has risen to limit the overtreatment, the related risk, and other important consequences. The idea of its adoption for low-risk thyroid cancer has been slowly developed since 1993. The number of its supporters gradually increases (88). However, the question of whether both physicians and patients are ready for it remains open. On the one hand, active surveillance may protect a significant number of patients from surgery with its potential complications and their treatment, life-long thyroid replacement therapy, or even from RAI administration and its consequences. On the other hand, active surveillance may be related to a potential disease progression or other disadvantages, including anxiety due to untreated malignancy or the possibility of non-compliance to follow-up protocol (89).

The majority of papers analyze the outcomes of different treatment methods and other issues outstanding from the medical point of view. In contrast, the studies evaluating patient participation in the decision-making process are rarer. The choice of treatment method more frequently depends on physician decisions than on patient preference. The analysis based on the SEER registry, including 1,319 PTC patients, demonstrated that 55.8% of persons felt they did not have any choice regarding RAI administration and presented a lower satisfaction with the treatment decision. Nearly 95% of patients whose physicians recommended such therapy, were treated with RAI. On the contrary, only 22.5% of patients received postoperative RAI therapy, if their physician recommended against this treatment (90). Sawka et al. reported the results of a prospective mixed-methods study of decision making on surgery or active surveillance for low-risk PTC < 2 cm in maximum diameter. Standardized medical information was provided for all patients. Next, patients were interviewed after making the decision. Ninety-four percent of 100 patients enrolled independently chose the management for their disease, whereas the remaining persons shared the decision with their physicians. Seventy-one patients chose active surveillance, 29—immediate surgery. PTMC was more prevalent in individuals choosing active surveillance compared to patients choosing surgery, 54.9% vs. 27.6%, respectively. The vast majority of patients (96.6%) opting for surgery had postsecondary education. On the contrary, 33.8% of patients who preferred active surveillance finished their education at the high school level. Nearly all patients (98%) were satisfied with the decision they made. Perceived risk of thyroidectomy or cancer, family history of thyroid cancer or other malignancies, family considerations, trust in physicians, and treatment timing concerning life circumstances influenced their decision (77). Korean scientists compared the impact so-called “usual care” with the “conversation aid” approach on the decision-making process concerning treatment options in a group of 278 PTMC patients. In total, 233 (84%) patients preferred active surveillance, whereas 53 (16%) individuals – thyroid surgery. Patients from the conversation aid group showed a higher probability of choosing observation than surgical treatment, 88.9% vs. 77.0%, respectively (91). These data clearly demonstrate the importance of the conversation between patients and health caregivers. The authors emphasized that conversation meant much more than information.

A widely accepted paradigm that early detection and treatment is related to a higher cancer curability is deeply rooted in patients’ minds. They are much less familiar with the term “overdiagnosis” or “overtreatment”. Regardless of the level of decision satisfaction in PTC patients choosing active surveillance, anxiety or emotional stress related to cancer diagnosis and resignation from surgery is reported by a majority of patients (77, 92, 93). It has been demonstrated that the so-called disease label may exert a meaningful impact on the patient’s decision. An online survey, completed by 1068 predominantly healthy responders, showed their preferences between a series of two hypothetical vignettes concerning incidental detection of a small thyroid nodule, varied on disease label (cancer, tumor, or nodule), treatment (active surveillance, or lobectomy), and risk of progression/recurrence (0%, 1%, 2%, or 5%). The cancer label played a crucial role in the patient’s decision, independent of proposed therapy and progression/recurrence risk (94). The role of the terminology used to define the disease was also confirmed by the results of other survey conducted in 550 Australian patients without a history of thyroid cancer. Total thyroidectomy was chosen more frequently when the term PTC was used to name the condition, compared to using papillary lesion or abnormal cells, 19.6%, 10.5%, or 10.9%, respectively (95). Similar data were reported by a discrete choice experiment also carried out in the Australian population. This study involved 2,054 participants, also without a history of thyroid carcinoma, who were randomly assigned to receive 1 of 2 groups differed with terminology used to characterize the condition: “cancer” or “lesion”. Patients may decide to choose between one out of three options: thyroidectomy, lobectomy, and active surveillance. If the condition was labeled with “cancer”, patients were ready to accept a higher number of adverse effects, life-long medications, calcium problems, and fatigue to avoid cancer-related death than persons which condition was called lesion (96). We agree that the terminology used by health caregivers may influence the anxiety or emotional stress related to the unfavorable diagnosis. However, we are not sure that we are allowed to use a softer term when a malignancy is diagnosed. To avoid unnecessary treatment and different psychological, physical or social consequences related to overdiagnosis, ATA does not recommend screening for thyroid nodules and a biopsy of small lesions. As it was mentioned above, Japanese doctors prefer patients to be clearly informed about the disease, prognosis and any potential risk. One should stress, ATA approach seems much acceptable for cancerphobic European patients than a Japanese one.

The terms “overdiagnosis” or “overtreatment” are raised not only by physicians or scientists. Also, patients may feel overdiagnosed or overtreated. Interesting data come from a qualitative study carried out in persons aged 21 to 75 years, in whom thyroid nodule known or suspected to be malignant was found incidentally and who questioned the treatment method proposed by their physician. Eighteen patients from this group decided not to intervene. The median of the follow-up was 40 months (range 1–88 months). Twelve out of these patients, despite significant anxiety about cancer progression, chose the observation based on understanding issues of precision in diagnostics, cancer behavior, surgical risk, medication use, and a low risk of cancer-related death. These patients felt unsupported both by medical professionals and friends. Importantly, seven patients said they wished they did not know the diagnosis, four were ambivalent, whereas only six patients were feeling glad they were informed about the disease (92). Valuable data were provided by analyzing the experience of active surveillance in Japanese patients from Kuma Hospital. Thirty-seven percent out of 243 patients rated their worry about cancer as occurring sometimes or more. Thirty-two percent reported that concern about cancer affected their mood somewhat or a lot, whereas, in 14% of patients, it affected their ability to carry out routine activities somewhat or a lot. The cancer-related worry was most potent when they found out about the diagnosis, and in 60% of patients subsequently decreased. Eighty-three percent of patients agreed or strongly agreed that choosing active surveillance was the best one they could make (93).

Quality of life shall always be taken into consideration when deciding on how to manage a patient. The evaluation of physical and psychological health is one of the outcomes of the MAeSTro study currently conduced in PTMC in South Korea. This assessment involved 203 patients choosing active surveillance and 192 individuals treated with surgery who completed questionnaires at least at two time-points. There were no differences between the groups regarding age, tumor size, TSH, thyroglobulin (Tg), and Tg antibodies level. Among persons, who opted for immediate surgery, 58 patients underwent total thyroidectomy, 133—lobectomy, 171—central lymph node dissection, and 4—lateral neck lymphadenectomy. Hypoparathyroidism and voice changes were diagnosed in 16.2% and 8.3% of patients, respectively. Regarding the active surveillance group, four patients resigned from observation due to other reasons than disease progression, mainly because of worry and anxiety. Only one patient from the active surveillance group showed disease progression. Significantly better psychological, physical, and overall health was observed during follow-up in patients who resigned from surgery (97). The risk of surgically related complications is also of great importance. All patients after total thyroidectomy require life-long thyroid hormone replacement therapy, whereas a considerable percentage of them had hypoparathyroidism or voice changes. The risk of permanent voice cord paralysis and permanent hypoparathyroidism at the Kuma Hospital were 0.2% and 1.6%, respectively (26). It is not surprising that the group managed by immediate surgery showed a higher risk of transient vocal cord paralysis, and transient or permanent hypoparathyroidism than the active surveillance group, 4.1%, 16.7%, and 1.6% vs. 0.6%, 2.8%, and 0.08%, respectively. The ratios of patients requiring L-thyroxine supplementation and those who had the local postoperative complications were also higher in operated patients (98). The study conducted in Argentina reported an even higher risk of postoperative complications, observed in 24.4% of patients, which became permanent in 9.6% of low-risk PTC cases who did not decide for active surveillance (99). At least 1 year following surgery, patients who needed prolonged calcium and vitamin D administration demonstrated a lower quality of global health, physical, role, and emotional functioning, or insomnia compared to patients not receiving the supplementation (100). Even PTMC patients subjected to less aggressive thyroid surgery, like lobectomy, reported a worse quality of life compared to patients who decided not to operate (101). Choosing between surgery and active surveillance in PTMC, one should remember that long-term outcomes are similarly excellent in both groups. What is even more important, delayed surgery in PTMC, based on the data presented earlier in this paper, is not related to a higher risk of distant metastases or cancer-related mortality. Both approaches are associated with emotional stress and anxiety. Besides, surgery, particularly total thyroidectomy still performed in PTMC patients, may lead to permanent complications in a relatively large number of patients. Thus, the quality of life may play a crucial role in the decision-making process.

Some other issues not related to the tumor features may influence the choice between surgery and active surveillance. In some cases, insurance status exerts an impact on the extent of treatment applied (55, 102, 103). The analysis of The American College of Surgeons’ National Cancer Database aimed to identify independent predictors of more intensive treatment used in PTMC patients. This study involved 190,298 PTMC individuals without nodal or distant metastases diagnosed preoperatively. The majority of patients (73.4%) from the analyzed group had private insurance. These patients were the least likely to be diagnosed with cancers showing high-risk features. On the contrary, uninsured patients more frequently had an extrathyroidal extension, lymphovascular invasion, positive surgical margins, and distant metastases. The differences between insured and noninsured patients were significant. Patients with private or public health insurance were more likely to have PTMC compared with noninsured ones. Regardless of less aggressive carcinomas, privately insured patients were more likely to be treated more extensively. Private insurance independently increased the probability of total thyroidectomy, lymphadenectomy, and postoperative RAI therapy (102). It seems indisputable that such disparities should be avoided. Valuable data come from the most recent analysis based on 34 semi-structured interviews with 12 surgeons, 12 endocrinologists, and 10 patients diagnosed with <1.5 cm PTC. Both surgeons and endocrinologists believed that overdiagnosis led to overtreatment. They considered overdiagnosis as a key issue. Biopsy, usually a reflexive or habitual action, in their opinion, was a critical point for further intervention (104). We share this opinion. When the FNAB result is positive, it usually starts the treatment process. However, the patients did not use the term overdiagnosis. In their view, the way from diagnosis to treatment seemed automatic and inevitable. Moreover, some patients and physicians prefer biopsy, regardless of the guidelines, to minimize diagnostic uncertainty. Although physicians were aware of possible overtreatment, they emphasized the difficulties in resignation from treating a diagnosed cancer. Total thyroidectomy seemed a reasonable treatment option for them (104). Similar scenarios are not rare in daily practice. We realize, changing a well-established paradigm is not easy and requires time.

Economic aspects are not less important than medical, psychological, or ethical issues. Lubitz et al. estimated that US$ 1.6 billion of the medical costs spent on thyroid cancer care in 2013 might increase up to 3.5 billion in 2030. The calculated cost includes diagnostics, surgery, adjuvant therapy for newly diagnosed patients (41%), surveillance of survivors (37%), and nonoperative death costs attributable to thyroid cancer care (22%) (105). A possible way to reduce these costs is to reduce the number of newly diagnosed very low-risk patients as well as the number of patients followed-up for a long time in referral centers (106). FNAB was related to a greater mean 12-month direct costs than observation of small incidental thyroid nodules < 2 cm in diameter, 542.47$ compared to 411.55$, respectively (107). Another way is to change treatment schemes to be equally effective but more cost-effective ones. Japanese experiences demonstrated active surveillance was more cost-effective than surgery (26). A total 10-year cost of active surveillance in patients without delayed surgery was 167,780 yen/patients, whereas in patients referred to immediate surgery, it was 794,770–1,086,780 yen/patient (108). Similar conclusions come from other reports. The non-surgical approach was more cost-effective than immediate surgery during the first 16 years after PTMC diagnosis and thereafter, regardless of patient age (< 40 and ≥ 40 years), complications, and progression rates (109). However, one may found some opposite data pointing on better cost-effectiveness of surgery compared to active surveillance in PTMC (110, 111). Australian data showed the cost of surgical therapy of 10,226 Australian dollars, whereas hypothetic active surveillance 756 Australian dollars per year. So, the cost of surgery corresponded to the cost of 16.2 years of active surveillance (111).

## Conclusions

To sum up, overdiagnosis of indolent low-risk PTCs is a global phenomenon leading to overtreatment in many cases without any beneficial effect on survival and patients’ well-being. Numerous clinical trials are needed to provide the data, fulfilling evidence-based medicine criteria necessary to change our routine clinical management in PTMC patients. We may expect substantial changes in the near future. The question is whether we, both patients and physicians, are ready for it?

## Author Contributions

JK: study concept, searching and literature review, writing the manuscript. AK, AK-B, KD-R, and MH-G: searching and literature review. MO-W and DH-J: literature review and writing the manuscript. BJ: study supervision, revision of the final version of the manuscript. All authors contributed to the article and approved the submitted version.

## Funding

This work was supported by the National Centre for Research and Development project under the program “Prevention practices and treatment of civilization diseases” STRATEGMED (STRATEGMED2/267398/4/NCBR/2015) and partially supported by The National Centre for Research and Development Grant No PBS3/B3/32/2015.

## Conflict of Interest

The authors declare that the research was conducted in the absence of any commercial or financial relationships that could be construed as a potential conflict of interest.
